# An unusual presentation of a nodular hidradenoma: A case report and review of the literature

**DOI:** 10.1016/j.amsu.2020.11.050

**Published:** 2020-11-23

**Authors:** Walid Bijou, Rabii Laababsi, Youssef Oukessou, Sami Rouadi, Reda Abada, Mohammed Roubal, Mohammed Mahtar

**Affiliations:** ENT Department, Face and Neck Surgery, Hospital August, 20’1953, University Hospital Centre IBN ROCHD, Casablanca, Morrocco

**Keywords:** Nodular hidradenoma, Case report, Skin tumor, Clinical presentation

## Abstract

Nodular hidradenoma is a rare benign adnexal tumor. It is most frequently encountered in the head and neck region, trunk, and extremities. This tumor exhibits a high recurrence rate, and an association with malignancy.Many names have been used to describe this pathology.We report the case of a nodular hidradenoma in a 30-year-old moroccan woman who presented with a 2-year history of a swelling in her right preauricular region. Histological examination revealed the typical appearance of a nodular hidradenoma. The tumor was excised and one year after the initial presentation, there was no sign of recurrence.

We emphasize the importance of wide surgical excision with appropriate margins to prevent local recurrence. A close follow up of the patients is recommended.

## Highlights

• Nodular hidradenoma is a rare benign adnexal tumor. Itis most frequently encountered in the head and neck region, trunk, and extremities.
• This tumor exhibit a high recurrence rate, and an association with malignancy.
• We emphasize the importance of wide surgical excision with appropriate margins to prevent local recurrence. A close follow up of the patients is recommended.

## 1 Introduction

Nodular hidradenoma is a rare benign adnexal tumor. Considerable controversy surrounds its histogenesis. It has been proposed that clear cell variations are of apocrine differentiation whereas only a minority of tumors are of true eccrine derivation [1]. It occurs as a solitary solid or cystic neoplasm.It is most frequently encountered in the head and neck region, trunk and extremities [2]. It affects all ages, most commonly females in the forth to the eighth decades of life [3].Recurrence, malignant transformation and metastatic spread have all been described in association with this tumor [4]. We report a case of a nodular hidradenoma of the face without evidence recurrence one year after surgical treatment.

A 30-year-old female patient, with no significant past medical or family history, presented at the department of ENT with a swelling in the right preauricular region, increasing gradually in volume over the last 2 years.There is history of serous discharge from the swelling.

**Fig. 1:**
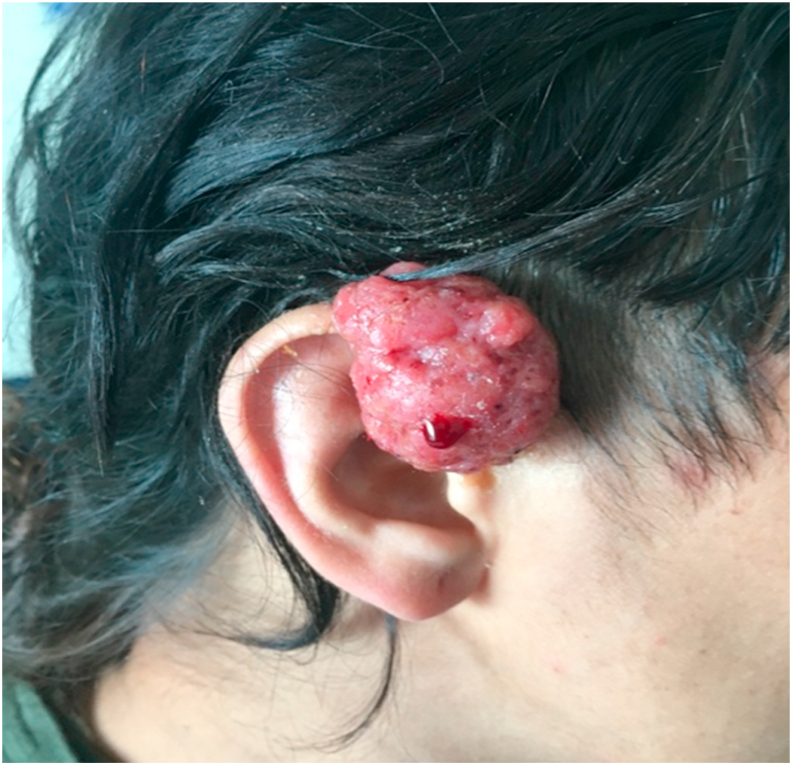
Patient at the time of presentation.

Physical examination revealed a reddish multilobulated pedunculated fleshy mass (4.0 × 3.0 cm) which bleedson touchin the right preauricular region, with an thickening of the overlying skin. The mass was freely mobile, non-compressible and non-pulsatile (Fig. 1). Regional lymphadenopathy was absent. Motor and sensory cranial nerve functions were within normal range. Prior to biopsy our clinical impressions were: Achromic melanoma, lymphoma, squamous cell carcinoma, Merkell cell carcinoma, metastasis, hidradenoma and cutaneous cylindroma.

The patient underwent an incisional biopsy of the massunder local anesthesia of the mass to exclude any malignant disease.

Histopathologic examination of the specimen revealed epithelial tumor proliferation with solid and cystic componentsmade of clear cells surrounded by myoepithelial cells. trabecular structures, were also seen in some areas.Rare cytonuclear atypia and mitosis patternswere noted. The histopathological pattern was consistent with a nodular hidradenoma.

**Fig. 2:**
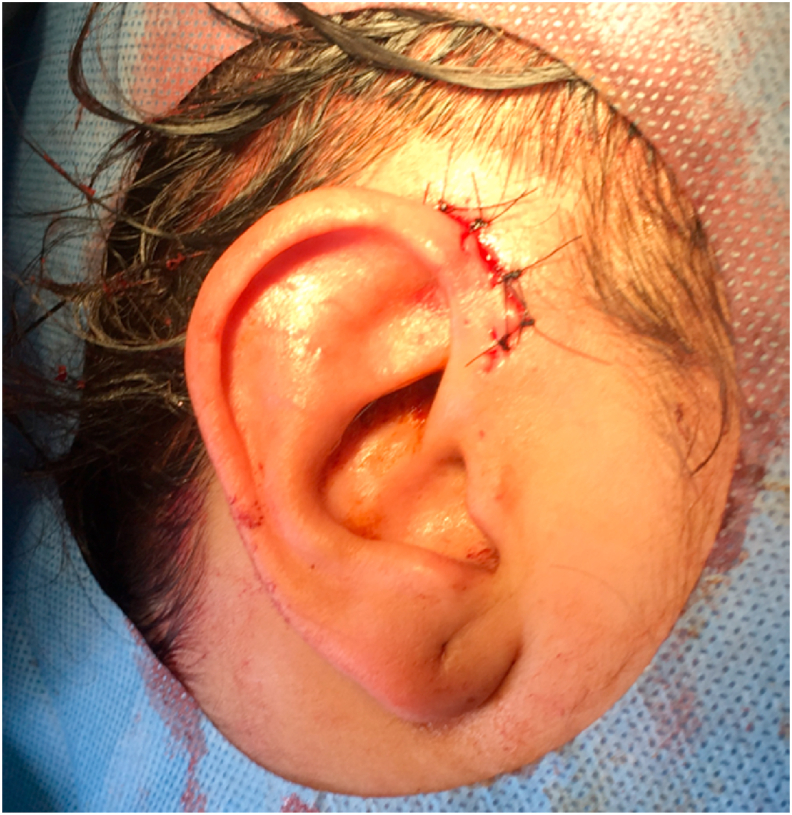
Post-operative view of the lesion.

**Fig. 3:**
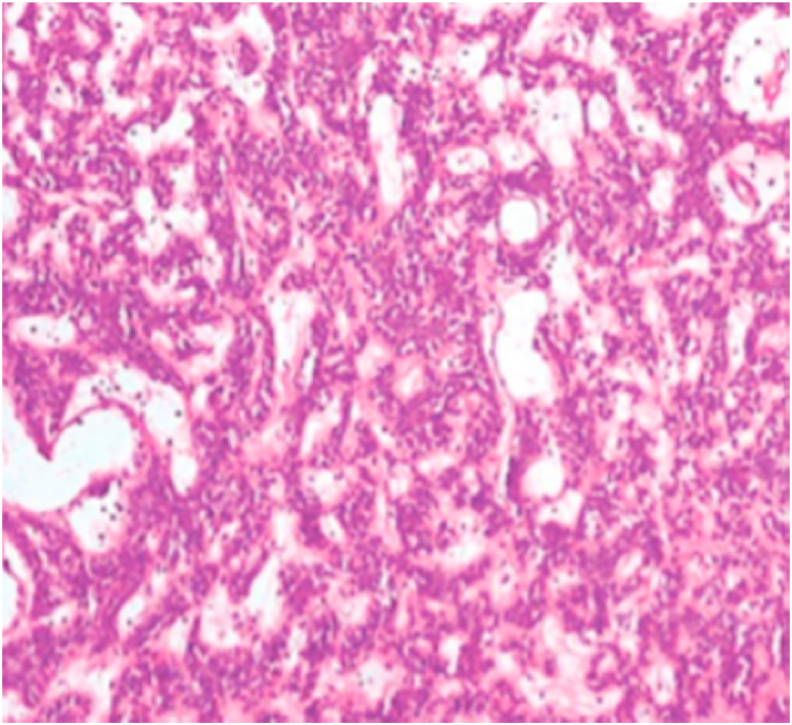
Cells with clear cytoplasm and small bland nuclei.

Therapeutic intervention: Surgical intervention was performed by our chief resident who has 5 years of operative experience. Local anesthetic of 4ml 2% lidocaine with 1 : 200,000 epinephrine was administered at the base of the tumor. Elliptical excision with wide margins was performed sharply using scalpel.After hemostasis with bipolar electrocautery, subcutaneous tissue and skin were sutured separately without tension using polyglactin and polypropylene interrupted sutures(Fig. 2). The postoperative period was uneventful (see Fig. 3).

The patient was reevaluated in the outpatient clinic of our department 1 month after discharge. There was no sign of facial nerve deficit.

Routine follow up 3, 6 and 12 months later showed no signs of recurrence.

This case has been reported in line with the SCARE criteria [5].

## 2 Discussion

Nodular hidradenoma is a rare benign sweat gland tumor. It is known under variable names [6]. (Table 1).The first reported case of nodularhidradenoma dates back to1949 [7]. Ohnishi et alhave proposed that clear cell variations are of apocrine differentiation whereas only a minority of tumors are of true eccrine derivation [1].

**Table 1:** Various terms used to describe this pathology.

Nodular hidradenoma
Clear cell hidradenoma
Solid-cyst hidradenoma
Eccrine acrospiroma
Clear cell acrospiroma
Eccrine sweat gland adenoma of clear cell type
Clear cell myoepithelioma

Nodular hidradenomas are usually seen in patients in their 40s–80s, with a pick incidence in the 6th decade [3].Exceptionally, children are affected [8]. Male to female ratio is1:2 [4]. This tumor is most frequently encountered in the head and neck region, trunk, and extremities (2).

Clinically, It appears usually as a small, solitary, nodular, superficial dermal lesion with intact overlying skin. Some tumors may exhibit ulceration on the surface or serous fluid leakage [3]. Our case was multilobulated andrelatively larger than average.

The differential diagnosis includes metastatic disease (renal cell carcinoma) and primary skin tumors with follicular differentiation, sebaceous differentiation, or sweat gland differentiation [9].

Histologically, Nodular hidradenomas may have variable patterns reflectedby the various terms used to describe this entity [3].It presents typically as a well-circumscribed unencapsulated tumor, located mainly in the dermis.The mass contains solid and cystic areas in varying proportions. The solid area presents 2 types of cells population: cellswith small dark eccentrically located nuclei with clear cytoplasm and round, fusiform, or polygonal cells with round or oval vesicular nuclei and eosinophilic cytoplasm [10]. The tumor cells express AE1/AE3, EMA, and CEA.Nonetheless, immunohistochemical analysis is not routinely required as most cases can be easily and reliably diagnosed with hematoxylin-and-eosin-stained sections [10].

Malignant transformation has been sporadically reported in the literature [4]. However, The exact incidence rate of transformation remains unknown. Criteria for assessing malignancy in sweat-gland tumors are the same as those used for other tumors:overt nuclear atypia, abnormal mitosis, infiltrative patterns, lymphatic or perineural invasion and areas of necrosis. Malignant hidradenomas express PHH3 >0.7% and/or Ki-67 > 11% [10].

Several cases are also described with deceptively benign histological appearances, but aggressive behavior, which make difficult thedistinction between hidradenocarcinoma and hidradenoma [3].

Recurrence of nodular hidradenoma is common, up to 10% [2], most probably due to inadequate excision of the tumor.The high recurrence rate and potential malignancy of this tumor highlight the need for adequate treatment. Complete excisionof the nodular hidradenoma with wide margins should prevent local recurrence. However, there is no consensus regarding optimal margins of excision in the literature.

House et al. [11] have suggested the use of theMohs micrographic surgery for recurrent or large hidradenoma, with encouraging results. Unfortunately, access to this technique can be difficult, especially in emerging countries.

## 3 Conclusion

Nodular hidradenoma is a rare skin adnexal tumorwith potential of aggressive behavior. We stress the importance of wide surgical excision with appropriate margins to prevent local recurrence. A close follow up of the patients is recommended.

## Ethical approval

Written informed consent was obtained from the patient for publication of this case report and accompanying images. A copy of the written consent is available for review by the Editor-in-Chief of this journal on request.

## Financial disclosure

The authors declared that this study has received no financial support.

## Author contributions

Walid Bijou: Corresponding author writing the paper

Rabii Laababsi: writing the paper

Youssef Oukessou: study concept

Reda Abada: study concept

Sami Rouadi: study concept

Mohamed Roubal: correction of the paper

Mohamed Mahtar: correction of the paper

## Provenance and peer review

Not commissioned, externally peer-reviewed.

## Declaration of competing interest

Authors of this article have no conflict or competing interests. All of the authors approved the final version of the manuscript.
